# High content analysis enables high-throughput nematicide discovery screening for measurement of viability and movement behavior in response to natural product samples

**DOI:** 10.1371/journal.pone.0205619

**Published:** 2019-04-23

**Authors:** Jennifer M. Petitte, Mary H. Lewis, Tucker K. Witsil, Xiang Huang, John W. Rice

**Affiliations:** Novozymes North America, Incorporated, Durham, NC, United States of America; Aarhus University, DENMARK

## Abstract

Historically, monitoring nematode movement and mortality in response to various potential nematicide treatments usually involved tedious manual microscopic analysis. High-content analysis instrumentation enables rapid and high-throughput collection of experimental data points on large numbers of individual worms simultaneously. The high-throughput platform outlined here should accelerate discovery of unique classes and types of promising lead molecules and sample types to control these plant pests. Also, the ability to automate the data analysis pipeline rather than relying on manual scoring reduces a potential source of data variance. Here we describe a high-throughput process based on high-content imaging. We demonstrate the use of time-lapse image acquisition to measure movement, and viability staining to confirm nematode mortality (versus paralysis) in targeted plant-pathogenic nematodes. We present screening results from a microbial-exudate library generated from approximately 2,300 microbial fermentations that demonstrate the robustness of this high-throughput process. The described methods should be applicable to other relevant nematode parasites with human, crop, or animal hosts.

## Introduction

Nematode infection of commercial crops is a wide-spread economic and food security issue resulting in yield loss worldwide[1]. Two main factors have hampered control of these crop pathogens: nematode populations have developed resistance to pesticides, and efficacious chemical pesticides have been removed from the market due to general environmental toxicity[2]. For these reasons, much of the research targeting methods of regulation of nematode infections has shifted focus to alternative control strategies that utilize naturally occurring / endogenous soil microbial and fungal strains [3,4].

Phenotypic nematode screening methods for active small molecules or microbes capable of controlling nematode infestations in both plants and animals can be as simple as manual microscopic examination of individual treatment samples or as complex as movement-based analysis of high-definition video captured worm images and microfluidics approaches [5–8]. All these approaches utilize visualization of some form, either manual scoring of non-movement vs. movement, or precise single worm movement measurements based on image analysis, such as “The Worminator” system [9]. Typically, plant-nematode screens are low-throughput and labor- intensive, and the observation of mortality is typically ascribed to an observed lack of movement, but is not distinguished from temporary paralysis or true mortality. We have developed a high-throughput, high content imaged-based assay approach that combines time-resolved image analysis for movement detection with the measurement of viable dye influx into affected worms, allowing for the collection of simultaneous movement and viability measurement data on an individual worm basis. This phenotyping assay method, which is capable of measuring data associated with tens of thousands of individual worms per day, coupled with the ability to test large sample libraries with automated liquid handling equipment, enabled screening of a proprietary microbial sample library to determine which individual microbe exudates display possible positive control effects on the tested nematode species. Here we present the details of an optimized high-throughput, time-lapse imaging and analysis platform for nematode phenotyping after exposure to microbial exudates. For this screening campaign, we targeted two widespread crop pests, the Root Knot Nematode *Meloidogyne incognita* (RKN) and the Soybean Cyst Nematodes *Heterodera glycines* (SCN).

## Materials and methods

Nematode samples were obtained from the laboratory of Dr. Rick Davis, located at North Carolina State University. Cultivar Williams 82 soybeans were from the laboratory of Dr. Mary Ann Quade, located at the University of Missouri, and cucumber seeds were cultivar Marketmore 76 obtained from JonnySeeds.com. PKH26 kits, octopamine HCL, and ivermectin were purchased from Sigma-Aldrich. Imaging CellCarrier plates were obtained from Perkin Elmer. SYTOX Green, brass sieves, and Breath-Easy plate seals were from ThermoFisher.

### Nematode culture

Nematodes were cultured on plant hosts growing in germination pouches within temperature-controlled growth chambers set for a 16/8-hour light/dark cycle and ambient humidity at 27°C. Soybean Cyst Nematode (SCN) eggs were harvested from soybean plants, cv. Williams 82, 4 weeks after inoculation of second stage juveniles (J2s) on 1-week old seedlings. Eggs were isolated from cysts using the standard graduated sieve system ranging from 850μm- 25μm pore sieve[9,10]. Captured eggs were placed on two layers of laboratory tissues supported by a wire screen sitting on a Petri dish filled with MilliQ (reverse osmosis, deionized water) water touching the bottom of the screen. This allowed hatched J2s to migrate through the laboratory tissue and fall into the petri dish. After an overnight incubation, J2s were collected to re-inoculate soybean plants or used in treatments. Root knot Nematodes (RKN) were cultured in a similar fashion from cucumber plants. For egg collection of RKN, root galls were minced and shaken for 3 minutes in a 0.25% sodium hypochlorite solution prepared in MilliQ water (v/v) and run through a series of sieves until final collection on a 25μm sieve. This assured complete rinsing and removal of residual sodium hypochlorite[11]. The RKN J2s were collected and used in the same fashion as described for the SCN.

### Worm bulk staining

For each batch staining of worms, approximately 100,000 J2 stage nematodes were used for both species. J2s were collected in MilliQ water and concentrated via gravity settling, and subsequently diluted to obtain no more than 15,000 J2s/mL. One milliliter aliquots of the resulting worm suspensions were centrifuged at 2,000rpm (400xg) for 5 minutes, followed by removal of the supernatant. PKH26 dye was prepared following the manufacture’s recommendations to a concentration of 30μM of dye stock per 1mL of buffer, and 1mL of the prepared dye solution was added to each tube of worms. Worms were resuspended by vortexing, followed by a 5-minute incubation in the dark. Following dye incubation, tubes were centrifuged remove the supernatant-containing dye buffer, and worms were washed 3 times via centrifugation in MilliQ water containing 1% bovine serum albumin (Sigma Aldrich).

### Microbial supernatant preparation and testing

All microbial broth sample preparations were performed in 24—or 96-well plate formats. A diverse set of ~2500 novel soil microbial isolates were grown for 3–7 days at 25°C. After growth, samples were centrifuged, and the supernatant was filtered through a 0.45μm filter. Samples were then stored at -80°C and assayed as described. For screening, 50μL of test sample was added to each well of individual assay plates using an Beckman FXp liquid handling robot. Each assay plate contained 12 negative control wells made up of MilliQ water only, 8 wells of NaOH controls (0.2% final concentration), 4 wells of ivermectin controls (10μM final concentration), and 4 wells of un-inoculated media controls (25% final concentration). All test samples were pre-diluted so that all final assays were performed at a 25% sample concentration. All samples were plated 3 separate times, allowing for all samples to be treated in triplicate. An example plate layout is presented in the supplementary data section (see S1 Fig.).

### Assay control reproducibility testing

Ivermectin stocks of 10mM were created by resuspending solid compound in DMSO. Test concentrations of ivermectin were made by dilution of this 10mM stock in MilliQ water. Testing of DMSO tolerance in the assay demonstrated no effect up to 10% total sample volume. NaOH test concentrations were prepared by dilution of 10% NaOH stock solutions into MilliQ water. Dose-response assays for ivermectin and NaOH were performed as described in the Microbial exudate treatment of worms section, each concentration was the mean of 8 wells of the individual dose responses on each plate, with error bars indicating standard deviation between the replicate plates.

For Z’-factor testing, each day of an experiment included 5 individual 96-well plates treated with control, 10μM ivermectin, and 0.1% NaOH, 32 individual wells per treatment plate. Plates were imaged 24 hours after treatment, 48 hours after treatment, and 6 days after treatment. For statistical analysis, Z’-factors were calculated for each individual plate and time point using the following formula:

$$Z^{\prime }=1-\left(\frac{3*SD\;treatment\;1\;mean+3*SD\;treatment\;2\;mean}{treatment\;1\;mean-teatment\;2\;mean}\right)$$

with control wells being used in all calculations for one of the treatment sets. To maximize worm exposure to treatment during microbial exudate treatment, and minimize variation introduced over treatment time, all final screening assays were performed using the 48-hour time point for data acquisition.

### Microbial exudate treatment of worms

After PKH26 J2 worm staining was completed, stained J2 worms were enumerated via microscopic examination and diluted in MilliQ water supplemented with 0.01% Tween 20 (v/v) to a concentration of 1 worm/μL. Stock formulated antimicrobial Penicillin/Streptomycin/Amphotericin B and SYTOX Green dye were added to the stained worm stock at a final concentration of 20,000 units/mL penicillin, 20μg/mL streptomycin sulfate, 50μg/mL amphotericin B (ThermoFisher), and 10μM SYTOX Green. Using a Multidrop (ThermoFisher), 50μL of the stained J2 solution was added to each well of a 96-well, black, clear-bottom view plate containing pre-dispensed test samples and controls. Slow and constant agitation of the J2 stock solution during dispensing assured equal distribution of J2s into the assay plates. Breathable plate seal membranes (Breath-Easy) were applied to each plate to reduce liquid evaporation during incubation. Plates were then incubated for 48 hours at 27°C. After treatment incubation, the Breath-Easy membranes were removed from the assay plates, and 10μL/well of an octopamine solution dissolved in MilliQ water was added to each plate with a Multidrop. For RKN, a 50mM octopamine solution was used, resulting in a final concentration of 5mM in the assay plate. For SCN, a 10mM octopamine solution was used, resulting in a 1mM final concentration in the assay plate. Before addition, the octopamine solutions were passed through a 0.2μm syringe filter. Following octopamine addition, plates were imaged within 24 hours on a GE IN Cell 2200 high content imaging platform. The IN Cell 2200 is an automated, high content analysis (HCA) microscope platform with epi-fluorescent capabilities, LED excitation, a high resolution sCMOS camera, with auto-focus capabilities that allows for rapid acquisition of sample images. Image acquisition was done using a 2X/0.1 Plan Apo objective, laser autofocus set to 1% energy, and sequential Cy3, FITC, Cy3 exposures. A 3-second delay was added between the FITC and final Cy3 exposure. All excitation exposures were 0.1–0.3 seconds duration, and all images were collected using a 2x2 bin. We typically imaged 20–40 plates per day, utilizing a Peak Automation KiNEDx automated arm to load and unload assay plates for imaging.

### Algorithm development and data reporting

An object detection algorithm was developed to analyze the collected images using the Cy3 signal from the worm cuticle stained with PKH26 to describe each individual worm, termed ‘object’. Area, length, and intensity thresholding was used to eliminate object detection noise, with values unique to each species. Motility was determined by analyzing the overlap of the object signal at time 0 and time 3 seconds. A worm that completely moved out of location was represented with a movement signal of 0, while a non-moving worm was scored as a 1. Each individual worm movement score was assigned a classification of “still” or “move”. “Still” was defined as a movement score of greater than 0.869 for RKN and greater than 0.9 for SCN. These defined values were assigned based on the movement inhibition exhibited by treatment with 10μM ivermectin. We observed that worms treated with high concentrations of ivermectin were still capable of slight movements during the 3 second time lapsed images, and the cut-off values for “movement” were assigned based on those observations. We were unable to increase the ivermectin concentration in the assay because precipitant formed at concentrations above 10μM, suggesting a limit to the compound’s solubility in water. There are reports in the literature from other laboratories that utilized imaging methods for worm analysis confirming the observation that ivermectin treatment rarely results in total movement inhibition, as well as the limited water solubility of ivermectin[8,12]. For the mortality measurement, SYTOX Green signal was analyzed in the FITC channel. We again used size and fluorescent-intensity gating to limit extraneous assay noise and background. Positive mortality (dead) was measured if a significant area of the worm, as defined by the PKH26, was colocalized with the SYTOX Green signal area. All algorithm settings are available in supplementary material (S2 and S3 Files).

After analysis, analyzed image data was output as well summary data with total worm counts for both time points, total count of dead worms, and total intensity levels for both FITC and Cy3 channels. Percent effect for each sample was calculated based on the percent of worms per well affected by the microbe exudate sample using a Python script for data analysis (S1 File). Final microbial effect was reported as an average of the well replicates based on the following formulas:

Raw (unadjusted) motility and mortality fractions were first calculated for each well as:

$$motility_{raw}=\;\frac{n_{move}}{n_{move}+n_{still}}$$

$$mortality_{raw}=\frac{n_{dead}}{n_{dead}+n_{alive}}$$

where *n* represented objects (worms) in the well. These raw fractions were then used to calculate the control-adjusted motility and mortality for each microbial sample:

$$sample_{adjusted}=\frac{sample_{raw}-mean\_neg\_control_{raw}}{mean\_pos\_control_{raw}-mean\_neg\_control_{raw}}$$

Controls specific to each analysis and subsequent calculations are shown in Table 1, with equations below. Note that motility expresses proportion of worms still moving, while mortality expresses the proportion of dead worms.

**Table 1:** Definition of specific controls used in this study.

	Motility	Mortality
**Negative control**	NaOH (dead = immobilized)	Untreated (live)
**Positive control**	Untreated (potentially motile)	NaOH (dead)
**Industry standard**	Ivermectin (likely immobilized)	

$$motility\_sample_{adjusted}=\frac{motility\_sample_{raw}-motility\_NaOH_{raw}}{motility\_untreated_{raw}-motility\_NaOH_{raw}}$$

$$mortality\_sample_{adjusted}=\frac{mortality\_sample_{raw}-mortality\_untreated_{raw}}{mortality\_NaOH_{raw}-mortality\_untreated_{raw}}$$

Adjusted motility and mortality scores were converted to percentages and averaged across replicate plates to provide final percent effect scores for each microbe sample.

As a final validation step, images of nematodes were manually scored and compared to scores achieved using the developed automated scoring process. To achieve mixed viable/non-viable samples, a pool of worms was heat-killed via treatment at 90°C for 10 minutes. This heat-killed pool was then mixed with a pool of control worms. Following the described methods for staining the worms, images were captured for 6 different sample wells containing ~50–70 worms per well. Statistical analysis comparing machine-scored and manual-scored worm phenotypes was performed in JMP version 14.01 (SAS Institute) resulted in a correlation value of 0.937 with a probability of <0.005, suggesting that the analysis methods returned similar results.

## Results and discussion

The assay we developed utilized two different fluorescent dyes, the lipophilic membrane PKH26 dye[13] and the viability dye SYTOX Green[14], which had been previously shown to stain non-viable nematodes[15]. By fluorescently pre-staining the nematodes with the permanent fluorescent marker PKH26, we believed we could produce high contrast images that would allow for the identification and localization of individual worms in treatment wells. Linking influx of SYTOX Green to each worm would then allow us to determine the viability of each individual in the treated population. Additionally, we found that by utilizing the time-lapse capabilities of our HCA instrument, along with image analysis, we could measure the movement of each worm at micron resolution. All the imaging was performed on the GE IN Cell 2200 instrument platform, a high content platform widely used in the human drug discovery area that allows cellular phenotypic analysis, individual object detection and segmentation, as well as cell-movement analysis[16–18].

**Fig 1:**
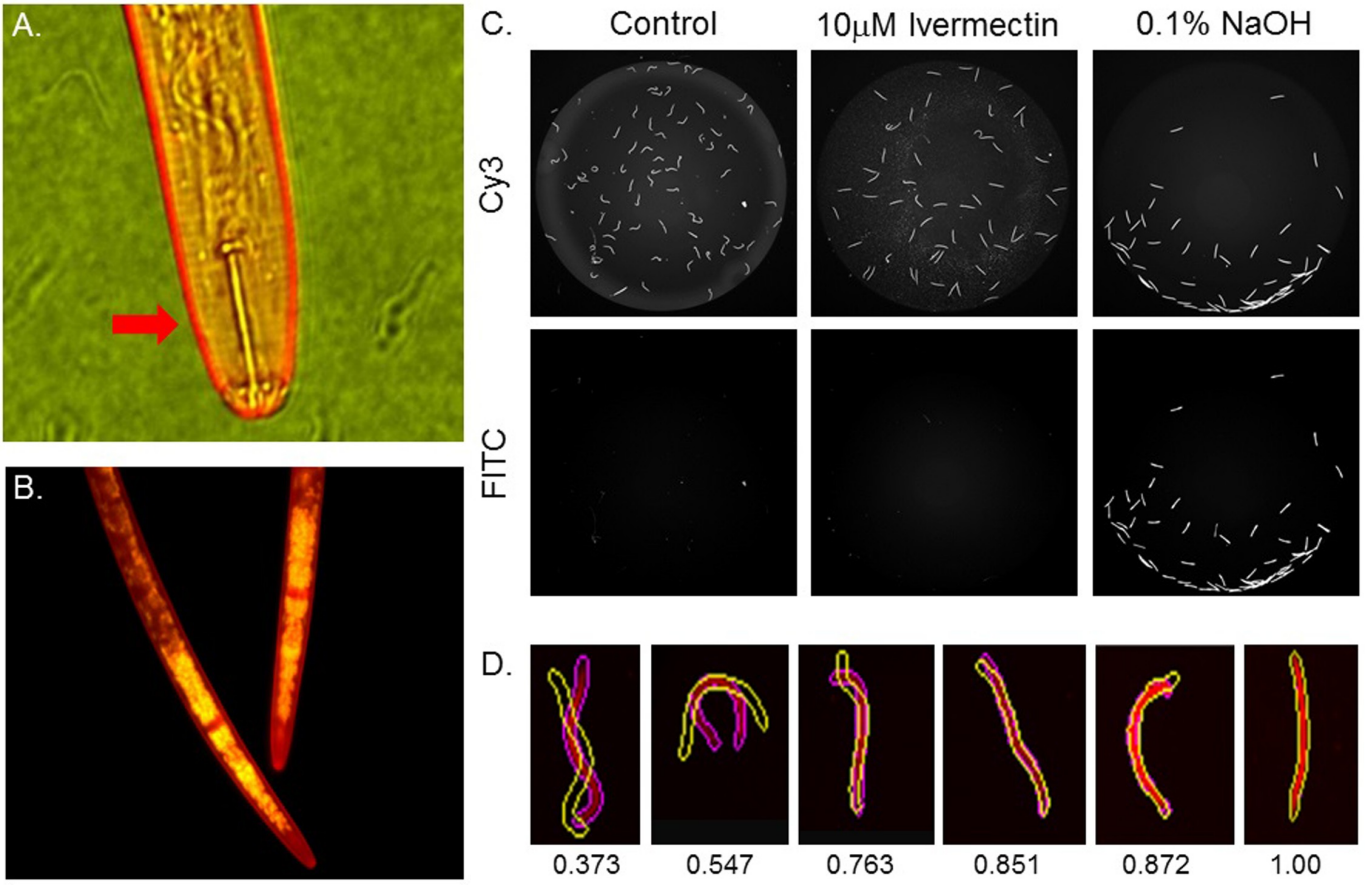
Imaging and quantification of nematode viability and movement. All images were obtained with a GE IN Cell 2200 HCA instrument. (**A**.) PKH26 lipid dye stains the outer cuticle of SCN, indicated by red arrow. (**B**.) A representative 60X merged image of heat-shocked RKN stained with both PKH26 (red) and SYTOX Green (green) showing internal DNA-binding of SYTOX Green after worm death. (**C**.) Representative RKN grey-scale images of PKH26 (Cy3) and SYTOX Green (FITC) treated with 10μM ivermectin and 0.1% NaOH. Only worms treated with 0.1% NaOH stain with the viable dye SYTOX Green. All intensity values were normalized between sample wells. (**D**.) Examples of Cy3- detected object outlines separated by a 3 second delay and overlaid by the GE Developer analysis software (time 1 = solid purple outline, time 2 = yellow outline). Movement is ranked based on 100% overlap (given a value of 1) and no overlap (given a value of 0). Fine differences are indicated by assigned score by the analysis software.

Assay validation experiments focused on several variables. First, the stability of PKH26 dye staining of the worms over multiple days in treatment plates and multiple imaging exposures was examined to assure minimal toxicity of the dye itself on the worms as well as minimal photobleaching of PKH26 when several exposures were performed. Secondly, worm distribution into 96-well test plates was optimized to assure accurate object detection and phenotyping by the software. Examples of un-treated control worms, non-moving worms resulting from ivermectin treatment, and NaOH-treated worms demonstrating both non-movement and SYTOX Green staining is presented in Fig 1. Since our goal was to identify microbe strains that exuded molecules capable of modulating the behavior of or killing the RKN and SCN nematodes, we included both ivermectin and NaOH controls in all screening plates. Using the GE IN Cell 2200, our final protocol measured SYTOX Green penetration in worms treated with 0.2% NaOH to identify worm death (Fig 1B and 1C), as well as time-lapse object overlay to validate inhibition of movement. We also included the well-characterized anthelmintic compound ivermectin[19] as a standard for characterization of paralyzed worms (Fig 1C).

Our assay conditions and detection algorithms could distinguish test samples capable of only paralyzing worms from samples capable of killing worms (Fig 1C). To assure we measured movement in all worms capable of movement responses, immediately before image capture the neurotransmitter octopamine was added to all sample wells to induce activity[20]. Each well of our 96-well assay plates had 3 separate images captured in the following sequence, Cy3 (PKH26 dye), FITC (SYTOX Green dye), and a second Cy3 image captured after a 3 second delay. The two Cy3 images were then overlaid using the colocalization function via the Cell Developer analysis software. We then created a custom detection algorithm within the GE Cell Developer software that allowed for the assessment of worm movement using the two sequential Cy3 images. The advantage of our HCA system and protocol was the coupling of high-resolution timed image capture with plate movement control and multiple fluorescent readouts resulting in a high-throughput phenotypic screening method with separate measurements for worm movement and worm mortality. Total worm movement could be quantified at single pixel resolution. In our case, at 2X magnification, that resolution was 3.25μm. Our final movement readout was based on the percentage of total overlapping pixels for individual worms, with each worm assigned a classification score by the analysis software (Fig 1D). Within each well, the percent of moving or still worms were then calculated. Final reported values were averages of replicate treatment wells.

**Fig 2:**
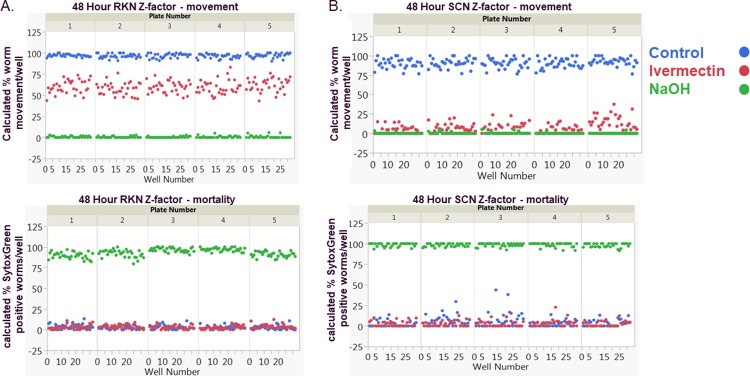
Representative data from a single day and time point of a 3-day set of experiments. Calculated average Z’-factors of the 3 days of 5 plates (n = 15 plates total representing 480 wells) for mortality based on 0.1%NaOH (**A**.), and movement for both 10μM ivermectin (**B.**). Images were captured at 24 hours, 48 hours and 6 days of treatment and all images transformed with Cell Developer software based on the algorithm described in the **Methods** section. Percent CV was calculated from the mean calculated Z’-factor for each individual day.

To demonstrate the reproducibility and accuracy of the selected controls, Z’-factors[21] were calculated for the differences between both positive-control treated worms and negative-control treated worms (Fig 2 and Table 2). Results from this Z’-factor test for average percent mortality and percent movement from NaOH-treated worms showed continued acceptable scores out to 6 days of treatment for both species tested. However, Z’-factor averages for both species treated with ivermectin demonstrated a continual decline in values that was time dependent, highlighting that both species were able to overcome ivermectin-induced paralysis. A Z’-factor greater than 0.5 is suggestive of a robust assay that is capable of identifying conditions that effect movement or mortality. Since NaOH could maintain an acceptable Z’-factor values for both movement and mortality, all calculated treatment values for screening were based on this control, ivermectin was included on screening plates for comparison only. Calculated Z’-factor results are presented in Table 2.

**Table 2:** Calculated Z’-factor testing results.

**A.** **Calculated percent mortality from 0.1% NaOH day-to-day Z’-factor**	**24-hour RKN**	**48-hour RKN**	**6-day RKN**
Ave. percent mortality	0.88	0.85	0.62
Standard deviation	0.04	0.07	0.33
Percent CV	4.90	8.04	52.83
	**24-hour SCN**	**48-hour SCN**	**6-day SCN**
Ave. percent mortality	0.85	0.76	0.41
Standard deviation	0.06	0.04	0.16
Percent CV	6.82	5.20	38.07
**B.** **Calculated percent movement inhibition from 10μM ivermectin day-to-day Z’-factor**	**24-hour RKN**	**48-hour RKN**	**6-day RKN**
Ave. percent movement	0.67	0.05	-2.71
Standard deviation	0.07	0.21	4.04
Percent CV	11.17	392.55	-149.16
	**24-hour SCN**	**48-hour SCN**	**6-day SCN**
Ave. percent movement	0.45	0.59	0.31
Standard deviation	0.14	0.05	0.22
Percent CV	31.66	8.20	69.78
**C.** **Calculated percent movement inhibition from 0.1% NaOH day-to-day Z’-factor**	**24-hour RKN**	**48-hour RKN**	**6-day RKN**
Ave. percent movement	0.91	0.89	0.71
Standard deviation	0.01	0.01	0.20
Percent CV	0.79	1.31	28.88
	**24-hour SCN**	**48-hour SCN**	**6-day SCN**
Ave. percent movement	0.75	0.74	0.79
Standard deviation	0.04	0.09	0.04
Percent CV	5.76	12.58	4.84

**Fig 3:**
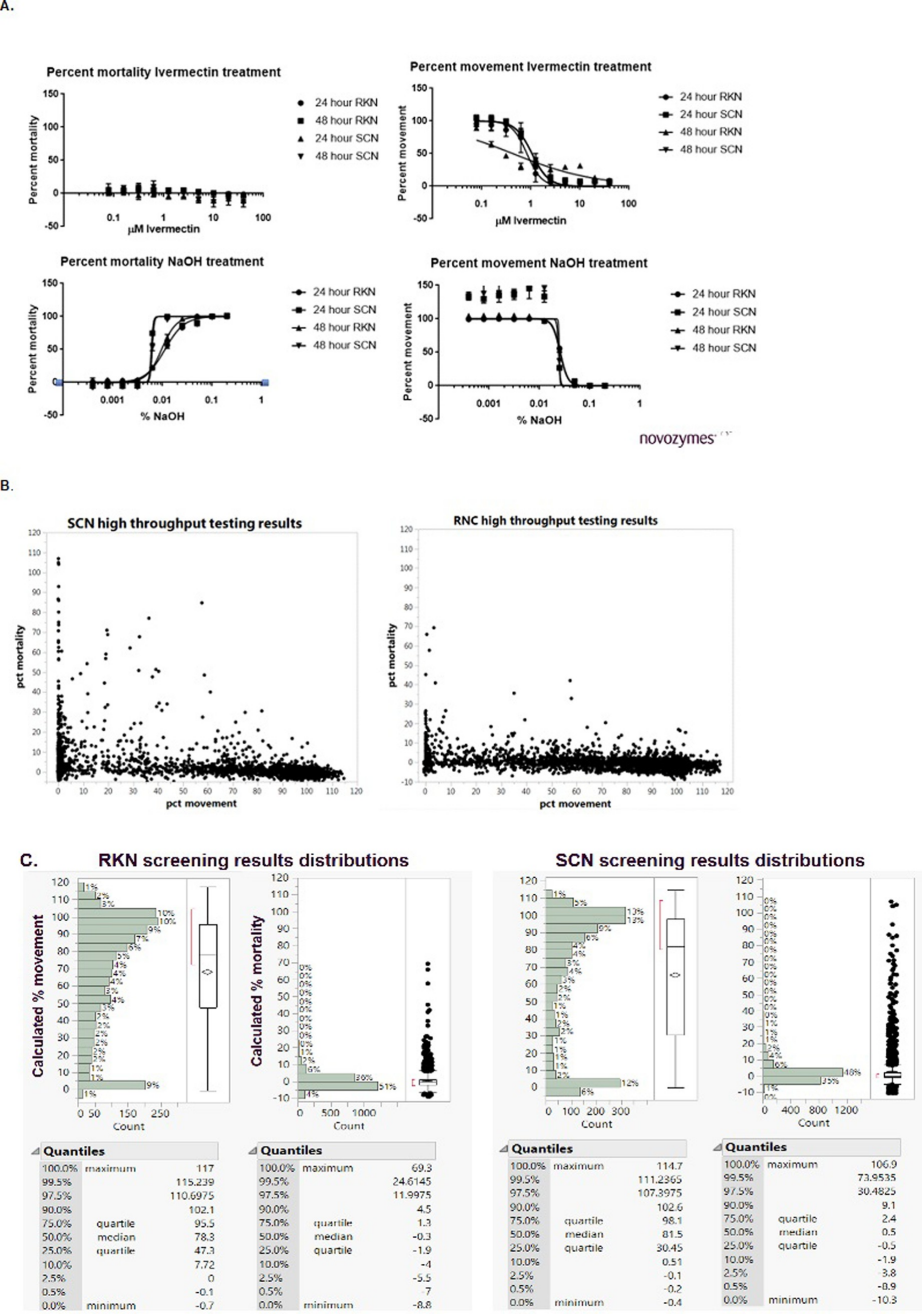
Assay accuracy and reproducibility. (A.) Demonstration of accuracy and repeatability based on 3 separate plates EC_50_ determinations for ivermectin and NaOH. (B.) Results from the high-throughput microbial exudate screening assays (C.) Distribution of exudate screening results.

Using our colocalization measurements on individual worms, we identified a dose-dependent range of movement for ivermectin-treated RKN and SCN. We used this scale to calibrate our paralysis- acceptability limits, as slight movement could still be seen in ivermectin-treated worms. Calculated EC_50_ movement values changed over time and were different for each species (Table 3 and Figs 1B and 3A). We did not observe any significant mortality associated with continuous 48-hour ivermectin treatment for any dose. Concentrations of ivermectin greater than 10μM resulted in fluorescent precipitation in the assay wells, reducing our ability to determine mortality effects with SYTOX Green above 10μM ivermectin. As a comparison, Ferreira *et*. *al*. reported the IC_50_ of ivermectin treated *C*. *elegans* for mortality as 261.4μM, but the EC_50_ for movement was 0.87μM, a ~300-fold difference in measured endpoints[15].

Upon close examination, we clearly have identified a concentration range of NaOH treated worms that displays some level of movement even with 100% levels of SYTOX Green-positive stained worms, as well as inhibition of movement with no concurrent increase in levels of SYTOX Green staining. This phenomenon can be discerned by comparison of the calculated EC_50_ values of NaOH-treated populations for both worm species (Table 3) where the difference between mortality and movement are approximately ~2-4-fold. Although widely used in both microbial and mammalian cell culture systems to determine viability, there are known discrepancies between true cell death and viability dye membrane penetration reported in the literature[22]. The complexity of a whole organism as opposed to a cell in culture also may be a large contributing factor to the noted discrepancy in viability and movement within the narrow range of NaOH dosing. This adds validity for the need to multiplex both a movement and mortality readout for a true indication of worm death. We also examined detergents such as Triton X -100 for possible use as positive controls but found the nematodes unaffected by up to 10% of detergent.

**Table 3:** Calculated EC_50_ values for ivermectin and NaOH.

**Percent Mortality**	**RKN 48-hours**	**SCN 48-hours**
Ivermectin EC_50_	**>40μM**	**>40μM**
Ivermectin EC_50_%CV	**NA**	**NA**
NaOH EC_50_ variable slope	**0.01%**	**0.006%**
NaOH EC_50_ std. error	**0.0002**	**0.24**
NaOH EC_50_ R square	**0.996**	**0.973**
**Percent Movement**		
Ivermectin EC_50_ variable slope	**0.43μM**	**1.06μM**
Ivermectin EC_50_ std. error	**0.088**	**0.048**
Ivermectin EC_50_ R square	**0.801**	**0.980**
NaOH EC_50_	**0.03%**	**0.02%**
NaOH EC_50_ std. error	**0.0004%**	
NaOH EC_50_ R square	**0.994**	**0.758**

To validate the reproducibility of the response to each treatment, dose response assays were performed in 3 individual 96-well plates for 48 hours. These tests were performed prior to sample screening. EC_50_ values were calculated with GraphPad Prism (version 7.04) software using Nonlinear regression curve fit, [inhibitor] vs. normalized response—variable slop. The mean value from the 8 replicate wells for each concentration on the three separate assay plates were used to create the 3 individual sets of data analyzed. As expected, we were unable to calculate an EC_50_ for ivermectin-driven mortality, as we detected little to no SYTOX green staining intensity. We were also unable to generate a calculated EC_50_ standard error for the NaOH-treated SCN at the 48-hour time point, but visual inspection of the curves highlighted very similar responses for the three separate treatment plates.

Because the images are a permanent data record of what is in each well at the time of image acquisition, we could compare manually-classified worm phenotypes from the images to machine-classified phenotypes and found a very high level of agreement (Table 4). We demonstrated that the machine count was predictive of the human counted results by applying a bivariate fit test, which returned a p-value of 0.0058 and an R-squared value of 0.8782. In addition, we also performed a correlation multivariate test on the machine and human-determined data and calculated the correlation coefficient to be equal to 0.9371, demonstrating strong correlation between the methods. This added validation to our developed automated detection algorithm and scoring scheme.

**Table 4:** Manual counting vs. machine counting results.

Treatment sample	Percent mortality detected automated algorithm	Percent mortality detected human counting
**Well #1**	**45**	**42**
**Well #2**	**49**	**61**
**Well #3**	**27**	**29**
**Well #4**	**54**	**57**
**Well #5**	**28**	**22**
**Well #6**	**57**	**57**

Based upon the robust statistically significant Z’-factor[14, 15] and reproducible EC_50_ results obtained for control treatments during our method development, we concluded that the automated image analysis protocol for nematode high-throughput screening described here is robust, reproducible and generates dependable data without variation inherent in manual worm observation (Figs 2 and 3 and Tables 2 and 3). In addition, the automated focus, robotic plate movement and stage movement of an HCA instrumentation pipeline provides the ability to screen large sample libraries in a short time.

As a final demonstration of the fitness of the described assay for high-throughput screening purposes, we prepared exudate samples from approximately 2,500 individual microbial isolates and tested these samples for the ability to influence nematode movement or mortality. Our results indicate a bias in our microbial library for strain exudates that affect movement at a level well above strains that influence worm mortality as demonstrated by Distribution analysis (Fig 3C). Treatment of RKN resulted in 10% of test samples demonstrate inhibition of movement below 5%, but <1% of tested samples demonstrate an increase in mortality using the modest mortality cut-off of >20%. Treatment of SCN resulted in 18% of exudate samples inhibiting movement below 5%, but only ~3% of tested samples show increased mortality >20%. Additionally, as expected, as percent movement drops, the number of strain exudates that cause a higher percent mortality measurement increases.

We typically screened up to forty 96-well plates at a time, each plate taking approximately 10 minutes to capture all images, for a total run time of 6.6 hours for 5760 individual analyzed wells. This sample throughput facilitates decreased timelines for sample library screening, discovery, and development of urgently needed anti-nematode chemistries or microbial inoculants that will help achieve the goal of reducing nematode-related crop yield loss and increasing food security. Additionally, it is reasonable to assume that similar approaches as those presented here should be applicable to both relevant animal and human nematode and related parasites. For example, with growing resistance becoming apparent in veterinary applications of ivermectin[23,24], additional animal health treatment options has become a necessity.

## Conclusion

The findings presented here indicate that the time-lapse capabilities of image analysis are useful for analysis of various biological samples, particularly for the quantification of movement. While nematodes were used in the assay methods we describe, with the goal of identifying microbial samples that inhibit movement or cause organism death, loss of movement of other organisms, or even single cells, in response to various stimuli should be amendable to similar assay protocols that will allow for complex multiplexed readouts. Instead of loss of movement, stimulation of movement should also be detectable. Our studies have also demonstrated that additional parameters (here, viability staining of the nematodes) can be used along with the time-lapse movement detection for a multiplexed assay readout allowing for confirmatory data, i.e. lack of movement equating to paralysis or organism mortality. Since high-content instrumentation is capable of detecting multiple fluorescent channels from the same samples, there also is the capacity to add additional metrics to multiplex the assay readout further and help elucidate additional biological complexity beyond the described endpoints.

We are aware of various image analysis software that is free and readily available for use such as Cell profiler and Worm Profiler (http://cellprofiler.org/, accessed 30NOV18) supported by the Broad Institute. Although not explored in our laboratory, this free-ware may also provide a suitable image analysis platform for experiments using our described time-lapsed method for worm movement detection. Certainly, any other commercially available analysis software should also be suitable for the described analysis, allowing for further adoption of the described processes.

Since the reason we developed the described assay was to test microbial exudate samples for biological activity resulting in plant parasitic nematode paralysis or, preferably, mortality, we undertook a screening campaign that examined ~2,500 unique samples generated from our proprietary microbial library. We completed a screening campaign consisting of 114 individual 96-well plates for both target pathogen species, with resulting control Z’-factor scores for all plates of 0.76±0.26, 0.83±0.10, 0.77±0.26, and 0.79±0.12 (average ± standard deviation) for SCN percent movement, SCN percent mortality, RKN percent movement, and RKN percent mortality respectively. These averages include assay plates that we ultimately failed for poor calculated Z’-factors, which we defined as a score in either control measurement below 0.5. This failure rate was ~10% of total assay plates, with all failures traced back to human error. We also were able to complete the actual screening over a 9-day period, highlighting the high-throughput capabilities of this HCA approach for screening modulators of plant pathogenic nematodes. Although we were unable to perform confirmatory assays to establish our sample reproducibility by repeating microbe fermentations for strains that displayed initial activity, we are encouraged by the final screening results and the apparent ability to differentiate activities between samples and look forward to further testing opportunities. Considering the complexity of our original biological sample test set, it would be very useful to understand our reproducibility rate and we look forward to performing those further studies.

As a final point, we realize that the idea of a multiplexed assay readout is not unique, any number of journal articles can easily be identified that describe various methods to measure relevant biological endpoints from a single sample to strengthen the relevance of the resulting data. In the past, we have used a high content, multiplexed approach to demonstrate heat shock protein 90 inhibition is responsible for multiple concurrent cellular responses, including the up-regulation of heat shock protein 70, the degradation of various phosphorylated proteins, and the inhibition of transcription factor translocation[25,26]. Recently, specialized imaging mass cytometry instrumentation was used to characterize cell and tissue samples by detection of protein, protein phosphorylation and RNA transcripts simultaneously[27] highlighting the power of multiplexed analysis approaches. High-content imaging methods are uniquely applicable to mammalian cell culture models, but we believe that by utilizing a similar multiplex assay readout approach, our ability to detect and identify samples capable of modulating the behavior or viability status of plant pathogenic nematodes has been greatly expanded as compared to the typical manual methods previously employed. We have demonstrated that the described high-throughput, multiplexed data output methodology allows for increased sample testing throughput, maximizing discovery opportunities to help identify microbes, natural products, or even small molecules capable of combating the loss crop yield caused by these specific nematode species. Finally, these described methods will hopefully prove to be robust enough to apply similar techniques targeting other worm pathogens of interest, including parasitic worms found in cattle and humans.

## Supporting information

S1 Fig Assay plate sample layout. A plate map describing the location of controls and samples in each assay plate used for microbial exudate sample screening.
(DOCX)

S1 Table Collated Z’-factor data. Data used for the calculation of Z’-factor scores. Experiments were run as described in Materials and Methods, with reported data representing the average worm movement and mortality scores for each individual treatment well.
(CSV)

S2 Table Screening control QA-QC summary. Calculated Z’-factor scores for screening plate controls.
(CSV)

S3 Table Plant nematode screening results. Results for microbial samples screened against plant pathogenic nematodes.
(CSV)

S4 Table Dose responses. Results from control testing dose responses.
(PZFX)

S1 File IN Cell_nematode_analysis_1.3. Python script used for screening data to generate final well results.
(PY) Click here for additional data file.

S2 File RKN detection algorithm script. Script generated in GE Developer software for the analysis of RKN movement and mortality.
(TXT)

S3 File SCN detection algorithm script. Script generated in GE Developer software for the analysis of SCN movement and mortality.
(TXT)

S4 File Hand determination vs machine determination. Data from a human count of representative worm images as compared to the data results from the detection algorithm for the same image set are presented.
(JMP)

S5 File Report correlation_hand vs. machine. Results of a correlation analysis between hand counted and machine counted worms from representative images is presented.
(JRP)

S6 File Report bivariate fit_hand vs machine. A final comparison of the hand counted worms and machine counted worms is presented.
(JRP)
