# Supplementary figures and images for: High content analysis enables high-throughput nematicide discovery screening for measurement of viability and movement behavior in response to natural product samples
Source: PLoS One. 2019 Apr 23;14(4):e0205619. doi: 10.1371/journal.pone.0205619 (PMC6478374; doi:10.1371/journal.pone.0205619)

**Assay plate sample layou**t:


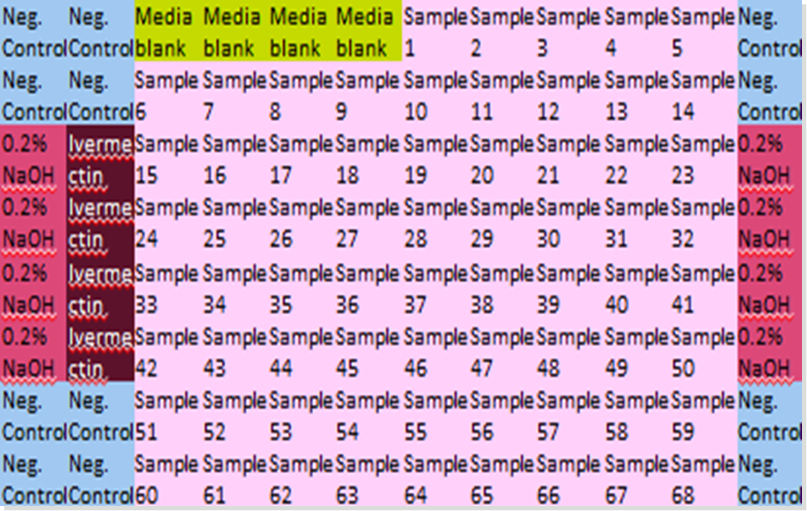

Supplement: S1 Fig — A plate map describing the location of controls and samples in each assay plate used for microbial exudate sample screening. (DOCX) [file pone.0205619.s001.docx]
